# *Ruminococcus gnavus* bacteraemia in a patient with multiple haematological malignancies

**DOI:** 10.1099/acmi.0.000048

**Published:** 2019-08-07

**Authors:** Caroline Gren, Malene Roed Spiegelhauer, Emelie Curovic Rotbain, Boje Kvorning Ehmsen, Peter Kampmann, Leif Percival Andersen

**Affiliations:** ^1^ Department of Clinical Microbiology, Rigshospitalet, Copenhagen, Denmark; ^2^ Department of Hematology, Rigshospitalet, Copenhagen, Denmark

**Keywords:** Microbiology, Bacteremia, *Ruminococcus gnavus*, hematology, MALDI-TOF MS, gut commensals

## Abstract

We present a case of *Ruminococcus gnavus* sepsis in a woman suffering from multiple myeloma and myelodysplastic syndrome. *R. gnavus *, a Gram-positive coccus and a gut commensal, has been described in nine cases of infection in the literature, with most infections having occurred in patients with either gastrointestinal symptoms or prosthesis infections. In this case, *R gnavus* was identified by mass spectrometry, and showed susceptibility to penicillin, meropenem, tetracycline, metronidazole and clindamycin. The patient was successfully treated initially with intravenous piperacillin/tazobactam and metronidazole, and then switched to oral penicillin and metronidazole. The cause of infection is hypothesized to have been a shift in the gut microbiota towards an excess growth of *R. gnavus* caused by immunosuppression, and bacterial translocation across a vulnerable mucosal barrier due to prednisolone treatment and severe thrombocytopenia.

## Introduction

*Ruminococcus gnavus* is a strict anaerobic Gram-positive coccus, which has been described as being part of the normal intestinal flora in humans [1]. To date, only nine cases of *R. gnavus* infections have been reported in the literature, predominantly in patients with gastrointestinal symptoms or prosthesis infection [2–9]. We present the first case of *R. gnavus* bacteraemia in a patient with multiple haematological malignancies, and with no abdominal complaint or prosthesis infection.

A literature review for articles describing clinical infections with *R. gnavus* was performed. Three databases were used for the search: REX (The Royal Danish Library, www.rex.kb.dk), PubMed (National Center for Biotechnology Information, https://www.ncbi.nlm.nih.gov/pubmed) and Scopus (Elsevier, www.scopus.com). The search was last repeated 13 May 2019.

Of the nine cases found, six involved patients with a background of gastrointestinal (GI) diseases such as diverticular disease, ulcerative colitis and cholecystitis [2–6], and one more was hypothesized to have had a GI fistula [7]. In four of the cases, the bacteria were found in a hip (native or alloplastic) [4, 7–9]. See Table 1 for further information on previous cases. The previously described isolates were sensitive towards most types of antibiotics tested (see Table 2).

**Table 1.** Existing literature on patients with *R. gnavus* infection

**Year**	**Country**	**Age (years**)	**Gender**	**Previous conditions**	**Symptoms/findings at admission**	**Sample**	**Reference**
2018	Denmark	76	Female	Multiple myeloma, myelodysplastic syndrome	Asymptomatic. No clinical findings. Increase in CRP	Blood	Present case
2018	Spain	77	Male	Multiple myeloma, sigmoid colon cancer. Received chemotherapy	Sleepiness, bone pain, low-grade fever, acute respiratory failure, hypotension, oliguria with acute kidney injury	Blood	[2]
2018	Belgium	66	Female	Fecal peritonitis, small bowel herniation and perforation	Not available	Blood	[3]
2017	Spain	72	Female	Hip implant	Dysuria, groin pain, fever	Granuloma/pseudotumour tissue and sonicate from the prosthetic implant	[8]
2017	Republic of Korea	82	Female	DM, cholecystitis and CBD stone treated with ERCP and stent	Fever, cough, abdominal pain. CAT with gall bladder perforation	Blood	[6]
2017	Spain	93	Female	IHD, type 2 DM, hip prosthesis	Groin pain	Pus and sonicate from the prosthetic implant	[9]
2015	France	62	Male	Ulcerative colitis, hip prosthesis	Hip pain, fever	Bone	[4]
2014	France	47	Male	Squamous cell carcinoma, suspected gastrointestinal fistula	Hip pain, fever	Joint fluid	[7]
2013	Denmark	67	Male	Disseminated lung carcinoma	Abdominal pain, neutropenia. Laparotomy showed perforated diverticulitis	Blood	[5]
90	Male	Diverticular disease	Abdominal pain, vomiting, fever. CAT with diverticulosis	Blood

CRP: C-reactive protein; DM: diabetes mellitus; CBD: common bile duct; ERCP: endoscopic retrograde cholangiopancreatography; IHD: ischemic heart disease; CAT: computerized axial tomography

**Table 2.** Antibiotic susceptibility for *R. gnavus* in this and previous cases

**Antibiotic**	**Resistance in this case**	**Resistant isolates/total no. of isolates in previous cases**
Amoxicillin	nd	0/2
Amoxicillin/clavulanate	nd	0/2
Benzylpenicillin	S	1/5
Cefalothin	nd	0/1
Cefotaxime	nd	0/2
Ceftriaxone^a^	nd	0/1
Clindamycin	S	2/6
Erythromycin	nd	1/1
Gentamicin	nd	1/1
Imipenem	nd	0/3
Levofloxacin	nd	2/2
Lincomycin	nd	1/1
Linezolid	nd	½
Meropenem	S	0/2
Metronidazole	S	0/7
Moxifloxacin^a^	nd	3/3
Piperacillin/tazobactam	nd	0/5
Pristinamycin	nd	0/1
Rifampicin	nd	0/2
Tetracycline	S	nd
Ticarcillin/clavulanate	nd	0/1
Tigecycline^a^	nd	1/4
Trimethoprim/sulfamethoxazole	nd	0/1
Vancomycin	nd	0/7

a No breakpoints were found for Gram-positive anaerobes, and the non-species-specific breakpoints were used.

nd: No data, S: susceptible.

The genus *Ruminococcus* was first described in 1948, and *R. gnavus* was described in 1967. Due to gene sequencing by 16S rRNA, some of the species have been reassigned to the new genus *Blautia *, part of the order *Clostridiales *. *R. gnavus* has kept its name despite reassignment to the new genus [5]. *R. gnavus* is a frequently found commensal of the gut. It has mucolytic activity due to glycosidase activity [10] and may therefore come into close contact with the intestinal epithelia. It also has beta-glucuronidase activity, which enables it to generate toxic and possibly carcinogenic metabolites and thus cause local inflammation [11]. A shift in the intestinal microbiota to an abundance of *R. gnavus* has been linked to several diseases, such as Crohn’s disease (CD) [12–14], pouchitis after ileocolonic resection in CD patients [15], *Clostridioides difficile* infection in patients with inflammatory bowel disease (IBD) [16], spondyloarthritis [17], respiratory allergy in infants [18] and eczema in infants [19]. In patients with IBD, a shift from the mucolytic *Akkermansia* to an abundance of *R. gnavus *, which also has mucolytic properties, has been observed. This shift in bacteria has been proposed as a biomarker for decreased mucosal integrity in IBD patients [20].

## Case report

The patient, a 76-year-old woman with both multiple myeloma (MM) and myelodysplastic syndrome (MDS), was seen in the haematological outpatient clinic for a planned blood transfusion. The patient had no history of GI disease apart from minor GI bleedings, and no implants or history of alloplastic surgery. The most recent surgery the patient had undergone was an operation for a rotator cuff lesion 7 years previously. Six days earlier she had received two red blood cell transfusions and one transfusion of pooled platelets. The patient was diagnosed with light-chain MM 7 years ago. During the following years she received several lines of treatment with alkylating chemotherapy, glucocorticoids, immunomodulatory drugs and radiation therapy. Two years ago, she developed transfusion-dependent anaemia and thrombocytopenia and a new bone marrow biopsy and cytogenetic karyotyping revealed therapy-related MDS. Treatment with 5-azacytidine and recombinant erythropoietin or thrombopoietin was ineffective. Subsequently the patient was put on supportive treatment with oral 12.5 mg prednisolone daily and supportive transfusion therapy. She was in partial remission for her MM at the time of infection. Due to severe thrombocytopenia and prednisolone treatment the patient had had several prior episodes with minor GI bleedings before the time of the infection. The patient had also previously been examined for infections several times before the time of *R. gnavus* infection. Sixteen days before the positive blood culture with *R. gnavus* was drawn, the patient was discharged from the hospital after an admission because of suspected tonsillitis. Blood cultures were negative, and the patient was found to be negative for influenza virus A and B and respiratory syncytial virus. No other microbiological tests were performed. The patient was treated empirically with intravenous piperacillin/tazobactam and was discharged with oral penicillin and pivmecillinam. Two months before the present case story the patient had diarrhoea and tested positive for *C. difficile* toxin and was treated with oral vancomycin for 2 weeks. Blood cultures drawn at that time were negative. Three months earlier the patient was treated empirically with oral amoxicillin/clavulanic acid because of an increased C-reactive protein (CRP) level of 79 mg l^−1^. No microbiological tests were performed at that time. The microbiological record of the patient contained no other prior positive results and contained two negative sets of blood cultures obtained respectively 10 and 12 months before the time of *R. gnavus* infection.

At admission, blood tests revealed an increase in CRP to 127 mg l^−1^ from 74 mg l^−1^ measured 6 days earlier. The CRP value had been elevated (>10 mg l^−1^) for 4 months. In this patient, as in many other patients with MDS, increased CRP levels are a paraneoplastic phenomenon. Her total leucocyte count at the time was 1.5×10^9^ cells l^−1^ and, as usual, she was severely thrombocytopenic, with fewer than 3×10^9^ platelets l^−1^. She had no complaints, and in particular no abdominal complaints. She had a normal physical examination, was afebrile with normal vital signs, and there was no evidence of current bleeding. Her medications were oral treatment with 12.5 mg prednisolone once daily and prophylactic tranexamic acid to minimize bleeding episodes.

Blood cultures were drawn, and the patient started antibiotic treatment with oral administration of ciprofloxacin 500 mg twice daily and amoxicillin/clavulanic acid 500/125 mg three times daily. She was discharged to an early follow-up appointment. She was contacted and admitted to hospital the following day when the blood cultures revealed Gram-positive cocci. The patient was still asymptomatic and afebrile. Ciprofloxacin and amoxicillin/clavulanic acid were discontinued, and she started continuous intravenous treatment with 14 g/24 h of piperacillin/tazobactam and one dose of 1 g vancomycin. Three days after admission, oral metronidazole 500 mg three times daily was added. Five days after admission, piperacillin/tazobactam was changed to oral penicillin 1 million international units (IU) three times daily based on the results of antibiotic susceptibility testing, and the patient was discharged the following day in her usual condition with oral treatment with penicillin and metronidazole for another 4 days. The patient was on antibiotic treatment for 11 days in total.

One set of blood cultures was performed, consisting of one aerobic and one anaerobic bottle (BD BACTEC, Becton Dickinson, Franklin Lakes, NJ, USA). The anaerobic bottle was positive at day 2, and a Gram stain showed Gram-positive cocci in chain form. A second set of blood cultures was obtained 1 day after the first set, and thus after the initiation of antibiotic therapy, and no bacteria were found. Two weeks after this event, another set of blood cultures was obtained, again without growth of pathogens.

The blood was prepared for analysis by matrix-assisted laser desorption/ionization time-of-flight mass spectrometry (MALDI-TOF MS) by collecting 1 ml blood in a tube (Eppendorf, Hamburg, Germany) containing 200 µl 7 % saponin lysis buffer (Sigma Aldrich, St Louis, MI, USA).

The sample was vortexed for 15 s, incubated at room temperature for 5 min and centrifuged for 1 min at 13.000 r.p.m. The supernatant was removed, the pellet was resuspended in 1 ml 0.85 % NaCl water and vortexed. The sample was centrifuged for 1 min at 13.000 r.p.m again and the supernatant was removed. The pellet was smeared on a target plate, and 1 µl of 70 % formic acid (Honeywell Fluka, Charlotte, NC, USA) was added. The plate was left to dry, and 1 µl of HCAA matrix solution (Bruker, Billerica, MA, USA) was added. Further identification was achieved on a Microflex LT mass spectrometer (Bruker, Billerica MA, USA). The generated spectrum was analysed using IVD MALDI Biotyper (version 3.4) and Bruker Taxonomy/Compass Library (version *7*) software. MALDI-TOF MS identified the pathogen as *R. gnavus* with a score of 1.78. The following three suggestions on the list of pathogens based on MALDI-TOF MS were also *R. gnavus *, but with lower scores.

The antibiotic susceptibility was tested for penicillin, meropenem, tetracycline, metronidazole and clindamycin by agar disc diffusion according to our laboratory’s standard procedure for Gram-positive bacteria. A suspension of the isolate with a concentration of 0.5 McFarland was plated on 7 % defibrinated horse blood agar plates (SSI Diagnostica A/S, Hillerød, Denmark). Antibiotic discs (Rosco Diagnostica A/S, Taastrup, Denmark) were added onto the plate, and were gently pressed down to the agar with sterile forceps. The plates were incubated for 24–48 h. The sizes of the zones were assessed using the European Committee on Antimicrobial Susceptibility Testing (EUCAST) clinical breakpoint system (version 8.0, 2018) for Gram-positive anaerobic bacteria. The non-species-related breakpoints were used when there were no breakpoints for Gram-positive anaerobes. The bacterium was susceptible to all of the tested antibiotics, and no resistance was observed. No E-test rendering minimum inhibitory concentration (MIC) values was performed. See Table 2 for a comparison of the susceptibility between this case and previous cases.

## Discussion

We present the 10th reported case of *R. gnavus* infection. Unlike in previous cases, the patient was asymptomatic and had no implants, alloplastics or likely GI focus of infection. She had never experienced any GI symptoms apart from the aforementioned small GI bleedings. Thus, we propose that the mechanism of infection might be bacterial translocation from the gut to the bloodstream, caused by a vulnerable mucosal barrier due to minor intestinal bleedings because of severe thrombocytopenia and prednisolone treatment. The patient had received transfusions 6 days earlier, but we consider that to be an unlikely way of acquiring a very rare bacterial disease. The history of frequent infections is most likely due to an immunocompromised state because of MM, previous antineoplastic therapy [21, 22], MDS [23, 24] and present treatment with glucocorticoids [25].

The patient in our case had at the time of infection been immunocompromised for a long time. There is evidence that the immune system and the gut microbiota affect each other [26]; for example, a depleted microbiota may lead to a decrease in neutrophil production and function [27], and it is possible that the microbiota can cause inflammation in an immunocompromised host [28]. On the other hand, a healthy gut microbiota contributes to the host mucosal barrier [29]. A theory might therefore be that there had been shifts in the gut microbiota in this patient, which could have increased the risk of bacterial translocation due to increased permeability and an increased load of bacteria that are normally considered to be commensals. Several of the previously reported cases had also been in an immunocompromised state [2, 5, 7], and it seems plausible that immunocompromisation is a risk factor for *R. gnavus* infection. At this point, it is not known precisely why *R. gnavus *, and not one of the many other gut commensals, is seen as an invasive pathogen in these immunocompromised patients.

MALDI-TOF MS is commonly used for the routine identification of clinically relevant bacteria at our hospital. The instrument utilizes an ionizing laser to vaporize the bacterial proteins, for which the weight and relative abundance are measured, and the results are used to create a unique spectrum for the bacterium. This species is compared to the spectra in a database of spectra from already known bacteria (reference) and identified by finding the most similar spectra [30]. The index score that is obtained at analysis is a statement of how well the spectra match. An index score above 2 is a sure identification of the species, while a score between 1.7 and 2 is a certain identification at genus level [30]. In this case, the identification of *R. gnavus* resulted in an index score of 1.78, which according to the guidelines is a safe identification at genus level but not necessarily at species level. The Bruker Taxonomy/Compass Library database contains six references for *R. gnavus *. The relatively low score of 1.78 could have been a consequence of the small number of references, or it might have been because the sample was not of the best quality. There is nothing in the literature on infections caused by other *Ruminococcus* species, and it is quite likely that the identification of *R. gnavus* in this case is correct. The isolate was not saved, as we do not routinely perform 16S sequencing after MALDI-TOF MS analysis at our laboratory.

In conclusion, this case underlines the importance of having a high index of suspicion for infections in patients with haematological malignancy and patients receiving glucocorticoid therapy, even when only subtle clinical or paraclinical findings are present. Furthermore, the importance of thorough microbiological investigation in immunodeficient haematological patients is also evident.
